# Biomarkers for immunotherapy in bladder cancer: a moving target

**DOI:** 10.1186/s40425-017-0299-1

**Published:** 2017-11-21

**Authors:** David H. Aggen, Charles G. Drake

**Affiliations:** New York-Presbyterian/Columbia University Medical Center, Hematology/Oncology, 177 Fort Washington Avenue, 6GN-435, New York, NY 10032 USA

**Keywords:** Bladder cancer, PD-1, Pd-L1, Immunotherapy, Atezolizumab, Nivolumab, Pembrolizumab, Avelumab, Durvalumab, Tumor mutation burden, Immune checkpoint inhibitor, Biomarker, Nanostring

## Abstract

Treatment options for metastatic urothelial carcinoma (mUC) remained relative unchanged over the last 30 years with combination chemotherapy as the mainstay of treatment. Within the last year the landscape for mUC has seismically shifted following the approval of five therapies targeting the programmed cell death protein (PD-1)/programmed cell death ligand 1 (PD-L1) axis. Notably, the anti-PD-1 antibody pembrolizumab demonstrated improved OS relative to chemotherapy in a randomized phase III study for second line treatment of mUC; this level 1 evidence led to approval from the U.S. Food and Drug Administration (FDA). The PD-1 antibody nivolumab also demonstrated an overall survival benefit, in this case in comparison to historical controls. Similarly, antibodies targeting PD-L1 including atezolizumab, durvalumab, and avelumab have now received accelerated approval from the FDA as second line treatments for mUC, with durable response lasting more than 1 year in some patients. Some of these agents are approved in the first line setting as well - based on single-arm phase II studies atezolizumab and pembrolizumab received accelerated approval for first-line treatment of cisplatin ineligible patients. Despite these multiple approvals, the development of clinically useful biomarkers to determine the optimal treatment for patients remains somewhat elusive. In this review, we examine key clinical trial results with anti-PD1/PD-L1 antibodies and discuss progress towards developing novel biomarkers beyond PD-L1 expression.

## Background

**Fig. 1:**
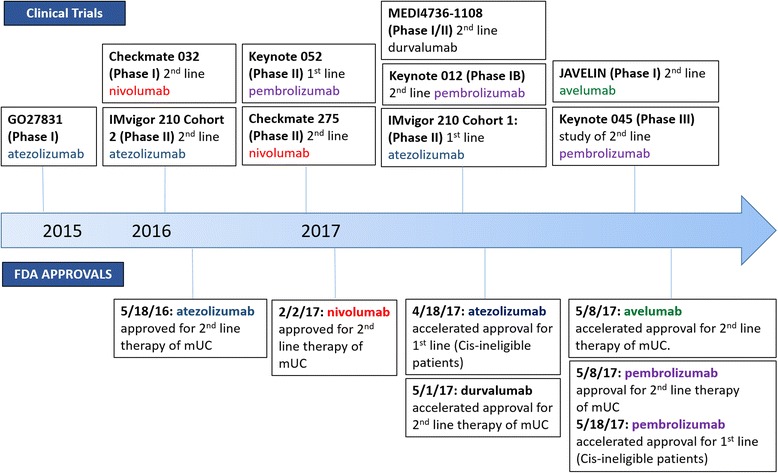
Timeline of clinical studies of programmed cell death protein/programed death-ligand 1 inhibitors in urothelial carcinoma

Approximately 79,000 new cases of bladder cancer are estimated in the United States in 2017, resulting in 16,870 deaths [1]. Worldwide, it is estimated that there will be ~168,000 deaths attributed to urothelial cancer in 2017 [2]. Although the majority of patients present with non-muscle invasive disease, approximately 30-40% of patients have muscle invasive disease at diagnosis with a worse prognosis. The five-year overall survival rate for all stages of urothelial cancer remains between 15 and 20%. Despite advances in treatment and survival, over the past 30 years treatment regimens for metastatic urothelial carcinoma remained relatively unchanged until the emergence of programmed cell death protein (PD-1) and programmed death ligand 1 (PD-L1) immune checkpoint therapies [3–5]. Within the last 18 months five new immunotherapies have been approved for second-line treatment of metastatic urothelial cancer (mUC) (Fig. 1). With this new armamentarium of treatment options focus has shifted toward developing novel biomarkers for treatment stratification. Here we review approved anti-PD-1 and anti-PD-L1 therapies and discuss future directions for combination immunotherapies. In addition, we highlight potential biomarkers to guide treatment decisions with particular attention to those that focus on the genetic level.

## Immunotherapy for metastatic Urothelial cancer

The five immunotherapy agents that are FDA approved for the treatment of metastatic urothelial carcinoma all have similar objective response rates (ORR) - between 15 and 23% in unselected patients in the second line setting (Table 1). Atezolizumab, nivolumab, durvalumab, and avelumab were approved based on single-arm studies comparing median overall survival (OS) and ORR with historical controls. Pembrolizumab is the only therapy validated in a randomized, phase III trial. A brief review of the clinical trial data that led to each approval follows.

**Table 1:** Response rates and median overall survival with FDA approved anti-PD-1/PD-L1 blockade in metastatic urothelial carcinoma

	Medication	Phase		# Patients	ORR (%)	PFS (m.)	OS (m.)	PD-L1 Response	Reference
Metastatic 2nd Line Therapy	*Atezolizumab*	I		100	21.0	–	8	–	Powles et al. [7]
		II		310	15.0	2.1	7.9	PD-L1 on IC > 5% associated with ORR, testing not required for treatment	Rosenberg et al. [8]
	*Pembrolizumab*	III	P	270	21.6	2.1	10.3	PD-L1 TC and IC composite score > 10%, no difference in ORR or mOS	Bellmunt et al. [13]
			C	272	6.7	3.3	7.4	–	
	*Nivolumab*	II		270	19.6	2.0	8.74	PD-L1 on TC > 1% not associated with ORR but associated with OS	Sharma et al. [11]
	*Avelumab*	Ib/II		241	17.6	1.6	7.0	PD-L1 on TC > 5% associated with improved ORR, no OS data as of yet	Apolo et al. [22] Patel et al. [23]
	*Durvalumab*	Ib		191	17.8	–	–	Composite biomarker of PD-L1 > 25% on TC or IC predicts response rates, approved companion diagnostic	Massard et al. [19, 21]
Metastatic 1st Line*	*Atezolizumab*	II		100	23.0	2.7	15.9	PD-L1 on IC not associated with improved ORR or mOS	Balar et al. [10]
	*Pembrolizumab*	II		370	29.0	–	–	PD-L1 TC and IC composite score with cutoff of 10%, no difference noted in ORR	Balar et al. [16]

*Cisplatin Ineligible Patients

## Atezolizumab

Atezolizumab, a humanized anti-PD-L1 IgG1 antibody engineered to minimize binding to Fc receptors, was the first therapy granted approval by the FDA [6, 7]. Approval was based on the IMvigor 210 study, a single-arm phase II trial in which mUC patients received 1200 mg of atezolizumab at 3 week intervals [8]. This trial had two cohorts; Cohort 2 of IMvigor enrolled patients who had disease progression during or following platinum-based chemotherapy or within 12 months of neoadjuvant or adjuvant therapy. Objective response rates in patients receiving atezolizumab were 14.8% (CI 11.1-19.3) for the entire study population, with an ORR of 9.5% noted in patients with low PD-L1 immune cell (IC) expression (<5%) and 26% in patients with high PD-L1 IC (>5%) immunohistochemistry (IHC). Of note, in this trial samples were assayed using the SP142 assay [9]. Based on a pre-specified response rate of 10% in historical controls, the FDA-granted approval for atezolizumab for patients who progress after platinum-based therapy or who have progressed within 1 year of neoadjuvant or adjuvant therapy on a platinum containing regimen. The median overall survival in patients receiving second line atezolizumab was 7.9 months (CI 6.7-9.3 m.). At a median follow-up of 11.7 months, responses were ongoing in 38/45 responding patients (84%), suggesting that a proportion of patients experienced sustained benefit.

The other cohort of this trial, Cohort 1, enrolled cisplatin-ineligible patients who were treated with first-line atezolizumab at an identical dosing schedule to cohort II [10]. The majority of patients in cohort 1 suffered from renal impairment that prohibited cisplatin based therapy (70%). The ORR for that cohort of 123 patients was approximately 23%, in comparison to a widely accepted historical control ORR of 10%. The median overall survival for the entire cohort was 15.9 months (95% CI: 10.4 m. – not estimable) with 21% of the patient population remaining on therapy for more than 1 year. In surprising contrast to what was observed in cohort II, responses in cohort I appeared to be independent of PD-L1 status (ORR of 28% vs. 21% for >5% PD-L1 IC expression and <5% PD-L1 IC expression). Median overall survival was also independent of PD-L1 status (12.3 vs. 19.1 months for >5% PD-L1 IC and <5% PD-L1 IC expression. Based on the favorable ORR relative to historical controls the FDA approved atezolizumab for use in cisplatin-ineligible patients with metastatic urothelial carcinoma.

The most common adverse events (AE) noted with atezolizumab at the prespecified dose level in cohort I and cohort II were fatigue, diarrhea, and pruritis with rare instances of autoimmune phenomena commonly associated with PD-1 therapy including elevation in liver enzyme tests (3%), pneumonitis (2%) and hypothyroidism (7%). Recently, the randomized phase III IMvigor 211 study evaluating atezolizumab in comparison to chemotherapy as second line treatment was announced to have failed to reach its primary endpoint of improved overall survival, independent of PD-L1 expression status. More extensive data on that trial were not available at the time of writing, but that unexpected outcome highlights the need for improved patient stratification beyond PD-L1 testing to select appropriate mUC patients for immunotherapy.

## Nivolumab

Nivolumab, a fully-human IgG4 anti-PD1 antibody hinge-modified to improve half-life, received accelerated approval from the FDA for second line therapy in previously platinum treated mUC. This approval was based on data from the checkmate 275 trial, a single-arm phase II study that enrolled 270 patients to receive nivolumab at 3 mg/kg every 2 weeks [11]. The PD-L1 assay used in Checkmate 275 measured PD-L1 tumor cell expression using the 28-8 antibody (Dako PD-L1 IHC kit, Dako North America, Carpenteria, CA, USA) and differed from that in the IMvigor 210 cohort which measured immune cell PD-L1 expression using a different diagnostic antibody and staining protocol. Objective response rates for the entire population approached 19.6% in comparison to a widely accepted historical control objective response rate of 10%. In contrast to what was observed with atezolizumab in cohort II of IMvigor 210, responses appeared to be independent of PD-L1 tumor cell expression (objective response rates of 28.4%, 23.8%, and 16.1% were noted for tumor cell PD-L1 expression of >5%, > 1%, or <1% respectively). Conversely, median OS was 11.30 months for patients with ≥1% PD-L1 expression in comparison to 5.95 months with ≤1% PD-L1 expression. This compared favorably with historical controls from meta-analyses of second line chemotherapy that demonstrate a pooled median overall survival of 6.98 months [12]. The most common adverse events noted with nivolumab were grade 3 or 4 diarrhea, with 18% (48 of 270 patients) experiencing a grade 3 or 4 AE. There were 3 on-study deaths attributed to treatment; one case each of pneumonitis, acute respiratory failure, and cardiac compromise. Based on the overall response rate and relative safety, nivolumab was approved in February of 2017 for treatment of platinum refractory mUC.

## Pembrolizumab

Pembrolizumab is a hinge-stabilized, humanized IgG4 anti-PD1 antibody that, like nivolumab, disrupts the engagement of PD-1 with its ligands PD-L1 and PD-L2. Of the FDA-approved antibodies blocking the PD-1/PD-L1 interaction, pembrolizumab is the only agent approved based on data from a randomized, phase III study [13]. Approval was granted by the FDA based on the open-label Keynote-045 study which randomly assigned 542 patients who had recurred or progressed after platinum-based therapy to investigator’s choice chemotherapy (paclitaxel, docetaxel, or vinflunine) or to pembrolizumab at 200 mg every 3 weeks. The median overall survival in the pembrolizumab arm was improved as compared to the chemotherapy arm (10.3 m., CI 8.0-11.8 vs. 7.4 m., CI 6.1-8.3 m., *P* = 0.002). Progression free survival was not improved versus chemotherapy; this has been observed in other phase III studies of PD-1 blocking agents [14, 15]. The ORR for the pembrolizumab treated patients was significantly higher than in the chemotherapy group (21.1% vs 11.4%, *P* = 0.001). Consistent with what was observed in cohort I of IMvigor 210 with atezolizumab and Checkmate-275 with nivolumab, overall response rate was similar between groups with low and high PD-L1 expression as measured by tumor cell (TC) and immune cell (IC) PD-L1 expression using the Dako assay and the 22C3 antibody. I.E. the ORR was 21.1% in the total population versus 21.6% in the group with PD-L1 score of >10%. The lack of correlation between response rates and PD-L1 combined score again demonstrates an unmet need for biomarkers for treatment selection. The median overall survival of the PD-L1 high composite score group (>10%) was 8.0 months (CI 5.0-12.3) with pembrolizumab in comparison to 5.2 months (CI 4.0-7.4) in the chemotherapy group. While pembrolizumab clearly provides a survival benefit relative to chemotherapy, higher PD-L1 expression was not associated with increased survival relative to the entire pembrolizumab treatment group. Grade 3 or 4 adverse events were less frequent in the pembrolizumab group (15% with pembrolizumab vs 49.4% in the chemotherapy arm). Similar to nivolumab the most frequently reported side effects were pruritus (19.5%), fatigue (15.0%), nausea (11.3%), and diarrhea (10.1%).

Pembrolizumab was also approved for use as first-line therapy in cisplatin ineligible patients in mUC based on early data from the phase II Keynote-052 study [10, 16]. Overall survival data have not yet been reported. However, overall response rate was 27% for the entire study population with PFS and OS rates at 6 months of 31% and 67% respectively. An exploratory end-point in this study was overall response rate in patients with a PD-L1 composite score of >10%; here the ORR approached 51% (*n* = 42) [16, 17]. In an effort generate a biomarker with a higher negative predictive value, an exploratory analysis was performed with an 18-gene expression signature designating a T-cell inflamed phenotype as assessed by Nanostring (described later) [17]. This companion assay has been validated in a small number of patients treated with pembrolizumab with metastatic melanoma, gastric cancer, and head and neck cancer [18] and showed an even better association with overall response than did PD-L1 expression.

## Durvalumab

Durvalumab, an FcR binding deficient anti-PD-L1 antibody, was approved in May 2017 based on a single-arm phase I/II study evaluating 61 patients with platinum-treated advanced UC [19]. Patients were eligible if they had disease relapse within 1 year of neoadjuvant chemotherapy. The overall response rate for the entire cohort was 31.0%. Response rates for patients with PD-L1 expressing tumor cells were 46.4% in comparison to 22% for PD-L1 negative tumors. Here PD-L1 staining was performed using Ventana SP263 assay [9]. To select patients for durvalumab, an interesting composite biomarker defined PD-L1 ‘positivity’ if **either** tumor cells (TC) or immune cells (IC) demonstrated ≥25% staining by IHC [20]. In contrast, a patient was considered PD-L1 negative if both tumor cell and immune cell expressed ≤25% PD-L1. Utilizing this new composite biomarker, patients with negative PD-L1 expression in tumor cells and immune cells had an ORR of 0% (0 of 14) in comparison to an ORR of 46% for patients positive for PD-L1 in either compartment. A recent follow-up analysis reporting on 191 patients treated with durvalumab reported an ORR of 17.8% with an enrichment in response rates for PD-L1 high patients (ORR 27.6% vs. 5.1%) [21]. The FDA approved durvalumab along with a companion assay in May of 2017 (Ventana SP263).

## Avelumab

The single-arm JAVELIN phase I study evaluated avelumab, an IgG1 anti-PD-L1 antibody that blocks the interaction between PD-1 and PD-L1 but not PD-1 and PD-L2. In an initial phase Ib study of unselected patients with platinum refractory mUC the ORR was 18.2% with a reported median OS of 13.7 months [22]. All patients in the initial phase Ib cohort of 44 patients developed an adverse event, with infusion reactions noted in approximately 20% of patients. At a primary end-point of 12-weeks there was a trend towards increased survival in high PD-L1 expressing patients, with ORR of 53.8% vs 9.0% in PD-L1 tumor cell high relative to low expressing tumors (cutoff 5%, Dako PD-L1 IHC kit, Dako North America, Carpenteria, CA, USA, Merck 73-10 monoclonal antibody) [9]. Recently, a pooled analysis of an additional cohort of 241 patients with platinum refractory UC demonstrated a confirmed ORR of 17.6% [23]. The median overall survival for the pooled cohort approached 7.0 months (CI: 5.6–11.1). Using a similar cutoff of 5% PD-L1 tumor cell positivity, the ORR were 25% versus 14.7% for high and low PD-L1 expression respectively, with 6% of patients having severe AEs. The most common AEs noted with avelumab in more than 10% of patients included infusions reactions (22.8%) and fatigue (12.0%), with 11.6% of patients experiencing an autoimmune adverse event and 1 treatment related death attributed to pneumonitis. Based on comparison to historical controls, the FDA approved avelumab for 2nd line treatment of platinum-refractory locally advanced or metastatic UC.

## Combination immunotherapy trials

Extrapolating from studies in melanoma [24] and NSCLC [25], multiple trials are now aimed at evaluating combination PD-1/CTLA-4 blockade. Preliminary data from the randomized phase I/II Checkmate-032 trial showed safety of the combination at 2 dose levels resulting in ORR of 38.5% and 26% at higher and lower doses of ipilimumab respectively (3 mg/kg ipilimumab and 1 mg/kg nivolumab relative to 1 mg/kg ipilimumab and 3 mg/kg nivolumab) [26]. Given the improved overall response rate of 38.5% with nivolumab (1 mg/kg) and ipilimumab (3 mg/kg) relative to the 26% ORR observed with nivolumab monotherapy, a phase III study is planned to assess efficacy of the combination therapy (Checkmate-901, NCT03036098). In patients who have progressed on nivolumab, a small cohort of patients has been challenged with ipilimumab/nivolumab with response rates noted in 10% of patients [27]. Similarly, a phase III trial with targeted enrollment of 525 patients is accruing using combination durvalumab and tremelimumab in comparison to standard of care first-line chemotherapy [28].

A number of on-going trials are evaluating novel targets in combination with PD-1 therapy, including traditional chemotherapy [29], intra-vesical BCG, IDO inhibitors such as epacadostat [30], CD27 [31], CD137, OX-40 [32], and CSF1-R [33] (Tables 2 and 3). Recent phase I efforts explored the safety of combination nivolumab and ipilimumab with other approved tyrosine kinase inhibitors; data demonstrating the safety of combined nivolumab, ipilimumab, and cabozantinib was recently presented across genitourinary malignancies [34]. As the number of combination trials continue to expand, pre-clinical and translational validation of these targets will be essential to select combinations with the highest likelihood of efficacy.

**Table 2:** On-going combination immunotherapy trials in urothelial cancer

Therapy	Number	Phase	Trial ID	Est. Completion
Nivolumab +/− Ipilimumab (CheckMate-032)	1150 70	I/II II	NCT01928394 NCT02553642	December 2018 September 2018
Atezolizumab + MOXR0916 (anti-OX40) +/− Bevacizumab	762	I	NCT02410512	August 2018
CPI-444 + Atezolizumab	534	I/Ib	NCT02655822	June 2018
Pembrolizumab + PLX3397 (CSF1R)	400	I/II	NCT02452424	July 2019
BMS-986106 (anti-LAG3) +/− Nivolumab	360	I/II	NCT01968109	October 2019
MK-7684 +/− Pembrolizumab	336	I	NCT02964013	March 2020
GSK3174998 (anti-OX40) +/− Pembrolizumab	264	I	NCT02528357	January 2020
Pembrolizuamb + Lenvatinib	250	Ib/II	NCT02501096	January 2018
Durvalumab + Epacadostat	185	I/II	NCT02318277	January 2018
Pembrolizumab + Ramucirumab	155	I	NCT02443324	June 2018
Nivolumab + Cabozantinib +/− Ipilimumab	135	I	NCT02496208	December 2017
Atezolizumab + Epacadostat	118	I	NCT02298153	November 2020
Durvalumab + (Olaparib or Vistusertib or AZD1775 or AZD4547)	110	I	NCT02546661	March 2019
Durvalumab + Tremelimumab + polyICLC (TLR3 agonist)	102	I/II	NCT02643303	August 2022
Pembrolizuamb +/− Acalabrutinib	75	II	NCT02351739	Summer 2017
Tremelimumab +/− Durvalumab	64	II	NCT02527434	October 2018
Ipilimumab + Enoblituzumab (anti-B7-H3)	59	I	NCT02381314	March 2018
Atezolizumab + B-701 (FGFR3 inhibitor)	48	Ib	NCT03123055	April 2017
Pembrolizumab + Vorinostat	42	I	NCT02619253	May 2018
Pembrolizumab + Docetaxel or Gemcitabine	38	I	NCT02437370	December 2019
Nivolumab + Enadenotucirev (oncolytic virus)	30	I	NCT02636036	June 2018
Avelumab + NHS-IL-12	30	I	NCT02994953	April 2018
Pembrolizumab + Paclitaxel	27	II	NCT02581982	March 2019
Nivolumab + IFN-γ	15	I	NCT02614456	December 2017

**Table 3:** On-going studies evaluating first-line therapies for metastatic urothelial cancer

Therapy	Number	Phase	Primary Endpoint	Trial ID	Estimated Completion Date
Atezolizumab + Gemcitabine/Carboplatin vs. Gemcitabine/Carboplatin (IMvigor 130)	1200	III	PFS/OS	NCT02807636	July 2020
Pembrolizumab +/− Platinum vs Gemcitabine/Platinum (Keynote 361)	990	III	PFS/OS	NCT02853305	March 2020
Durvalumab +/− Tremelimumab vs. Gemcitabine/Carboplatin (1:1:1)	525	III	PFS/OS	NCT02516241	July 2019
Pembrolizumab + CVA21 (Coxsackievirus A21)	90	I	Safety	NCT02043665	August 2019
Nivolumab + NEO-PV-1 (personalized peptide vaccine)	90	Ib	Safety	NCT02897765	December 2020
Pembrolizumab + sEphB4-HSA	60	II	OS	NCT02717156	November 2020
Gemcitabine/Cisplatin +/− Ipilimumab (Active, not accruing)	36	II	Safety/ORR	NCT01524991	November 2017
Atezolizumab +/− Gemcitabine Cisplatin (First line metastatic or MIBC)	30	I/II	Safety	NCT02989584	December 2020

With the continued success of PD-1 targeted therapies in the metastatic setting a number of studies are evaluating immune checkpoint blockade in BCG-refractory non-muscle invasive bladder cancer. Early phase clinical trials evaluating BCG in combination with both pembrolizumab [35] (NCT02324582, NCT02808143) and atezolizumab [36] (NCT02792192) are currently accruing. It remains in open question if the potential autoimmune side effects related to immune checkpoint blockade will offset the potential benefits of PD-1 blockade in non-muscle invasive disease.

## Biomarkers for PD-1/PD-L1 blockade in mUC

### PD-L1 Expresssion

Expression of PD-L1 has been noted in between 20 and 30% of urothelial cancer specimens [37, 38]. More important, PD-L1 expression as measured by IHC in bladder tumors is associated with increased pathologic stage at resection and increased all-cause mortality, suggesting that high levels may indicate more aggressive disease [37, 39]. These data show that PD-L1 expression is **prognostic** in terms of outcome, a factor that needs to be taken into account when considering its **predictive** power in the context of PD-1 / PD-L1 targeted treatment. In urothelial cancer, phase II and phase III studies evaluated endpoints related to PD-L1 expression. PD-L1 staining assays and clinical results varied significantly across clinical trials in mUC highlighting the difficulties in using PD-L1 as a single biomarker. Data range from a strong association with overall responses using a composite biomarker that is required for patient selection (durvalumab [19, 21]) to no association as was noted in IMVigor Cohort 2 (atezolizumab 2nd line [8]), Keynote-045 (pembrolizumab [13]), and Checkmate-275 (nivolumab [11]).

One potential reason for these discrepancies is the use of 4 distinct assays for PD-L1 IHC scoring. For instance, pembrolizumab and nivolumab clinical trials use the Dako assay with the 22C3 and 28-8 antibody clones respectively. In contrast, durvalumab and atezolizumab use the Ventana assay and SP26 and SP142 antibody clones respectively [40]. In the setting of NSCLC, the four available anti-PD-L1 diagnostic assays have been compared with a greater degree of variability noted in PD-L1 IHC on immune cells [9]. In contrast, IHC of PD-L1 on tumor cells was comparable between the 22C3, 28-8, SP263 assays, whereas the SP142 assays showed significantly fewer PD-L1 positive tumor cells [14]. Another comparison of the 22C3, 28-8, SP26, and SP142 antibodies in 90 NSCLC specimens confirmed that SP-142 detected significantly lower mean PD-L1 TC and IC expression - again illustrating the challenges of comparing PD-L1 expression between assays [41]. In addition to inter-assay variability the scoring compartment differs for each specific therapy. Studies with pembrolizumab and nivolumab use PD-L1 tumor cell (TC) expression, whereas the IMVigor trials with atezolizumab use PD-L1 immune cell (IC) expression. In trials with durvalumab in mUC, a composite end-point was used as described above with 25% TC or IC expression delineated as high PD-L1 expression [20]. Aside from these technical challenges the PD-L1 tumor and immune cell status may not reflect the meaningful PD-1/PD-L1 interactions necessary for predicting a T cell response. Intratumoral heterogeneity may further limit the utility of PD-L1 IHC due to incomplete sampling and differential expression of PD-L1 not adequately represented in the biopsy specimen. Analysis of PD-L1 expression in whole tissue sections of NSCLC noted a discordance approaching 25% based on the section selected for IHC [42]. Finally, PD-L1 tumor status fails to account for PD-1/PD-L1 interactions that may be occurring in the tumor draining lymph nodes. Perhaps most importantly, PD-L1 as a tumor marker is dynamic over time and space and a single biopsy may not reflect the local effects on the cytokine milieu or the immune landscape in its entirety. Taken together it appears unlikely that PD-L1 as a stand-alone biomarker will achieve sufficient positive or negative predictive value to effectively guide treatment decisions.

## Clustering by TCGA subtype

Exploratory analyses in several trials retrospectively correlated The Cancer Genome Atlas (TCGA) urothelial cancer subtype with response to PD-1 / PD-L1 directed immunotherpy [43]. In cohort II (post chemotherapy) of the IMvigor210 study, TCGA classification was used to group patients into luminal (*n* = 73) or basal (*n* = 122) subtypes. Enrichment in PD-L1 immune cell expression was noted in the basal subtype (60% vs 23%), while tumor cell PD-L1 expression was noted almost exclusively in basal subtypes (39% vs 4%). Responses to atezolizumab were documented in all subtypes with a statistically higher response rate noted in luminal cluster II subtype (ORR = 34%, *p* = 0.0017) relative to luminal cluster I, basal cluster I, and basal cluster II (ORR 10%, 16%, and 20% respectively). A similar trend was noted in Cohort I of IMvigor with atezolizumab with the highest percentage of responses noted in the luminal cluster II group (*n* = 11/37, 7 partial responses and 4 complete responses). The TCGA subtypes were also an exploratory endpoint in the phase II Checkmate-275 study of nivolumab; by contrast here basal I subtype tumors represented the highest proportion of responders (7/23, ORR 30% for basal I). Luminal cluster II tumors treated with nivolumab had an overall response rate of ~25%. The reasons for these discrepancies in the mUC subtype most likely to respond might be related to tissue source. Both cohorts of IMVigor210 and Checkmate-275 allowed biopsy specimens from primary tumor, lymph nodes, or metastatic lesions for TCGA subtyping which may lead to inappropriate tumor classification. Second, the criteria for molecular subtyping differs in each study, highlighting a challenge in standardizing TCGA classification. Taken together these results are consistent with the notion that TCGA subtype is not likely to prove a strong predictive biomarker, especially across agents.

## Tumor mutational burden/Neoantigen burden

**Fig. 2:**
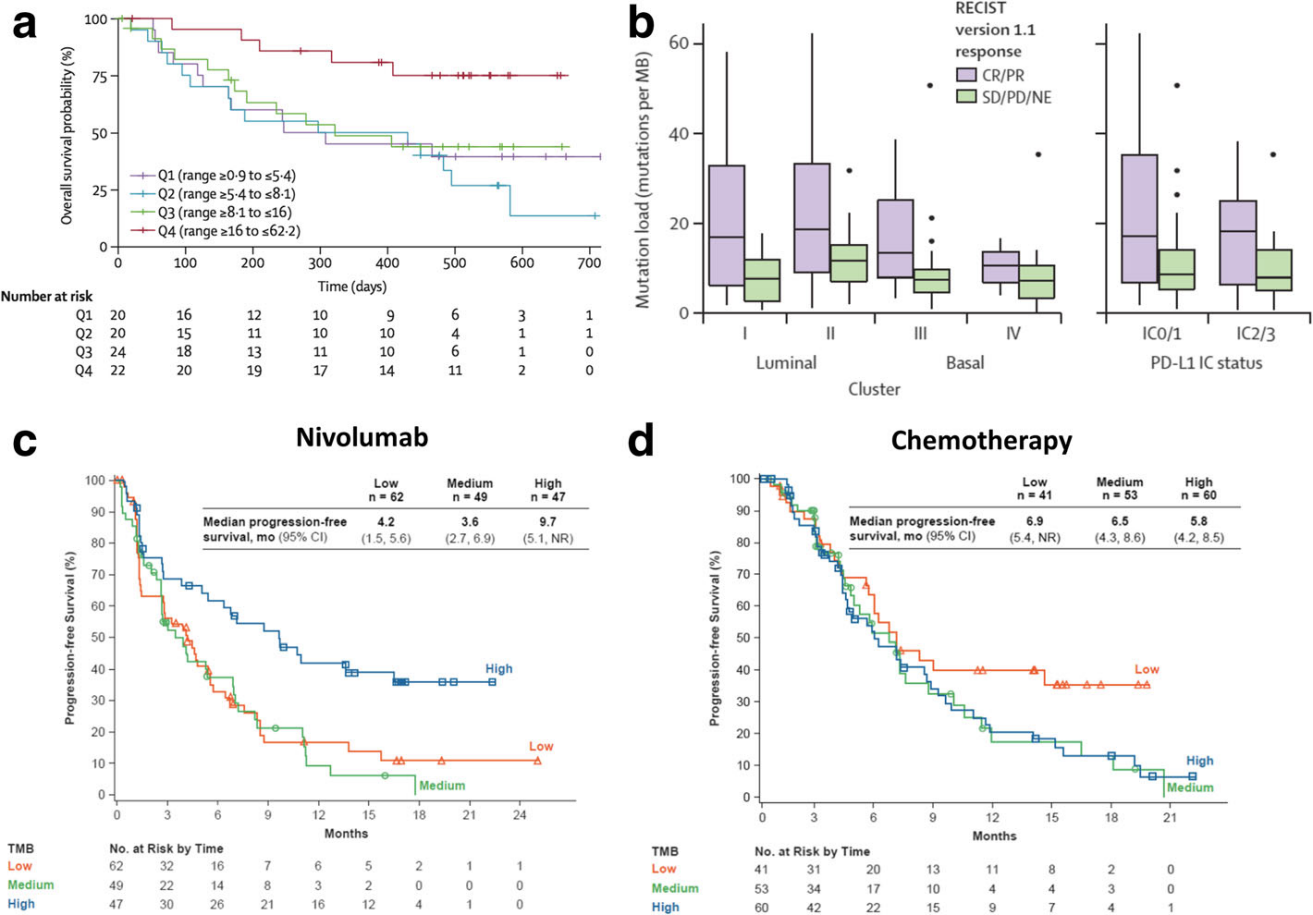
Tumor mutation burden as a biomarker for anti-PD-1/PD-L1 therapy. **a** Kaplan-Meier estimate of overall survival according to estimated mutational burden by quartiles in mUC patients treated with atezolizumab in IMVigor 210 – Cohort I. Range estimates next to each qauartile indicated number of mutations per megabase for each quartile. **b** Quantification of mutation burden across TCGA subtypes and PD-L1 immune cell IHC status and correlation with disease status. **c** and **d** Progression-free survival based on tertile of tumor mutation burden from Checkmate 026, a randomized study of nivolumab (**c**) compared to standard of care chemotherapy (**d**). A and B reprinted from *The Lancet,* Vol. 389, Balar et al. “Atezolizumab as first-line treatment in cisplatin-ineligible patients with locally advanced and metastatic urothelial carcinoma: a single-arm, multicenter, phase 2 trial, p. 73, 2017 with permission from Elsevier [10]. C and D reproduced with permission from Carbone, D. et al. *NEJM*. 2017., [48]

In both melanoma [44] and NSCLC [45], mutational load, as well as the number of predicted neoantigens is associated with a greater likelihood of durable responses to immune checkpoint blockade. In fact, those retrospective data suggest that mutational load may potentially predict response more robustly than PD-L1 IHC, presence of tumor infiltrating lymphocytes, or clinical variables [45]. While neoantigens have been identified by exome sequencing and validated with T cell activation assays - relatively few shared neoantigens have been identified and most neoantigens are likely to be patient specific [46–48]. As a consequence, a high non-synonomous mutation burden may correlate to an increased number of neoantigens and data from focused exome sequencing has demonstrated a correlation between tumor mutation burden (TMB) and response rates to immunotherapy. An exploratory subgroup analysis of IMvigor210 Cohort II evaluating 315 cancer related genes showed a significant increase in mutation load in responding relative to non-responding patients (12.4 per megabase vs. 6.4 per megabase, *P* < 0.0001) [8]. Smoking status and TCGA subtype did not correlate with mutational burden in a subset of 150 patients from IMvigor Cohort II, suggesting that TMB may be a more reliable predictor of response to PD-L1 blockade in urothelial cancer. Similarly, in cohort I of IMvigor 210, 119 tumor specimens were analyzed to determine tumor mutation load [10]. There was a clear correlation toward improved overall survival in the highest quartile of TMB (>16 to <62.2 mutations per megabase) relative to quartiles 1-3, with a Kaplan-Meier estimated survival probability approaching 75% at 1 year (Fig. 2a). The effect in improvement in overall survival was independent of TCGA subtype, with responses noted in all four TCGA subgroups (Fig. 2b). Of note, these data appear to suggest the possibility of a threshold effect, with patients in the lowest 3 quartiles appearing similar, whilst the top quartile seems to have an increased likelihood of responding and an overall survival benefit. The use of mutation burden to predict responses to immunotherapy was also highlighted in an exploratory analysis from the prospective checkmate-026 study evaluating nivolumab in first-line therapy for NSCLC. Checkmate-026 randomized patients with metastatic NSCLC and >1% PD-L1 expression to platinum-based chemotherapy or nivolumab [49, 50]. An exploratory secondary end-point assessed progression free survival (PFS) based on tumor mutation burden for ~50% of the study population who had tumor tissue available [51]. Patients with low or medium mutation burden status receiving nivolumab had inferior PFS relative to patients receiving standard of care chemotherapy (Fig. 2c and d). In contrast, patients with high levels of mutational burden responded favorably to nivolumab with improved median PFS, suggesting that high tumor mutation burden may predict responses to immunotherapy. Again, a threshold effect seemed to be present, with the lowest 2/3rd^s^ showing a similar RR and the top 1/3^rd^ deriving a progression free survival benefit.

Other studies used retrospective data to evaluate the relationship between the number of non-synonomous mutations and immunotherapy responses. Data in NSCLC using targeted exome sequencing of cancer specific genes identified an association between high mutation burden and durable overall response [52]. A composite score of PD-L1 status >1% and tumor mutation burden above the median correlated with response, with 60% of patients with high PD-L1 and high TMB deriving durable clinical responses. In a separate review of previously published data evaluating responses in melanoma and NSCLC, a threshold of 192 non-synonomous mutations (nSM) was hypothesized beyond which the the response rate to immunotherapy plateaus [53]. Using a cutoff of 192 nSM a 74% sensitivity and 59.3% specificity was identified to discriminate a potential benefit. The **negative** predictive value of TMB, however, is unclear, as responses were observed in some patients with low mutation burden.

Significant challenges confront the use of TMB as a predictive biomarker for immunotherapy. First is the challenge of unifying and standardizing the definition of mutation burden. For instance, some assays standardize for the size of the genome covered by targeted sequencing on a per megabase level. Others report based on absolute mutational burden which may fail to represent the true tumor mutation burden relative to the depth of sequencing performed. Second, gene fusions, truncations, and translocations may not be adequately covered by targeted sequencing panels and the value of these genetic events relative to single nucleotide variants in predicting response to immunotherapy remains to be determined. Third, germline variants may not be silenced by informatics techniques that filter common germline single nucleotide polymorphisms. As a consequence, uncommon germline variants may artificially increase the calculated tumor mutation burden, which highlights the need to improve standardization between tumor mutation burden assays. The somatic mutation burden is also likely to change dependent on other variables through the treatment course such as prior chemotherapy treatment and a biopsy at a single time point may not adequately reflect the relative antigenicity of the tumor. Despite these limitations there is now strong evidence that TMB correlates with durable responses to PD-1 blockade in multiple tumor types and with further standardization TMB will likely be a reliable surrogate to predict immunotherapy response.

Other surrogate measures of mutation burden such as chronic carcinogen exposure (eg. tobacco, ultraviolet light, aniline dye), defects in DNA repair mechanisms such as microsatellite instability/mismatch repair defects, and POLE mutations have emerged as potentially useful clinical biomarkers [45, 54]. Based on this notion and data demonstrating an overall response rate of 39.6% with pembrolizumab in microsatellite instability (MSI) high and mismatch repair deficient malignancies, pembrolizumab has been approved for patients with these gene defects solid after progression on prior treatments prompting investigation in a phase III setting [55–57]. This landmark FDA indication represents the first approval based on a tumor biomarker independent of tumor cell origin. Interestingly, certain mutational variants may portend a lack of benefit with PD-1 therapy, such as individual mutations in EGFR and STK11 that are associated with a lack of benefit in NSCLC and lung adenocarcinoma [52, 58].

**Fig. 3:**
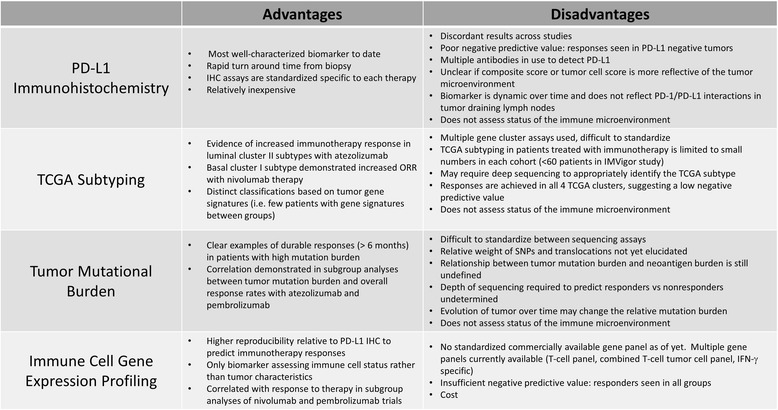
Advantages and disadvantages of potential biomarkers for immunotherapy

Composite variables integrating PD-L1 expression, TCR sequencing/TCR clonotypes, epigenetic analysis, and tumor mutation burden may delineate characteristics that predict responses to immunotherapy due to inherent advantages and disadvantages of each biomarker as a stand alone test (Fig. 3). These individual modalities are reviewed extensively elsewhere [59–61].

There are now numerous examples across solid tumor types including head and neck squamous cell cancer, NSCLC, melanoma, and urothelial cancer exploring correlation between composite markers and response to anti-PD1 [62, 63]. In mUC, whole exome sequencing, RNA sequencing, and T cell receptor sequencing were performed on pre and post-treatment biopsies of patients treated with atezolizumab to evaluate predictors of durable disease control [64]. In a small cohort of 24 patients a combination biomarker of elevated PD-L1 immune cell staining and high TCR clonality pretreatment were associated with poor clinical outcomes. In the same cohort mutation load was evaluated with different methodologies including missense mutational load, predicted neoantigen load, and expressed neoantigen load. All of these additional methodologies failed to demonstrate any association with control of disease for 6 months, highlighting the need to standardize these assays and develop improved composite biomarkers, which may ultimately hinge on the use of gene expression signatures.

Ongoing efforts evaluating combined measurements of mutational load with gene expression signatures show promise. Gene expression profiling performed in longitudinal tumor biopsies showed dynamic changes in multiple genes after the initiation of PD-1 therapy [65]. As these immune signatures are refined there is the potential that on-treatment biopsies may guide treatment decisions based on immune cell gene expression rather than based on imaging. To date, exploratory subanalysis have looked at larger, less well-validated panels in an attempt to better define an optimal immune signature using high numbers of reproducible gene transcripts.

## Multiparameter immune gene expression profiling

**Fig. 4:**
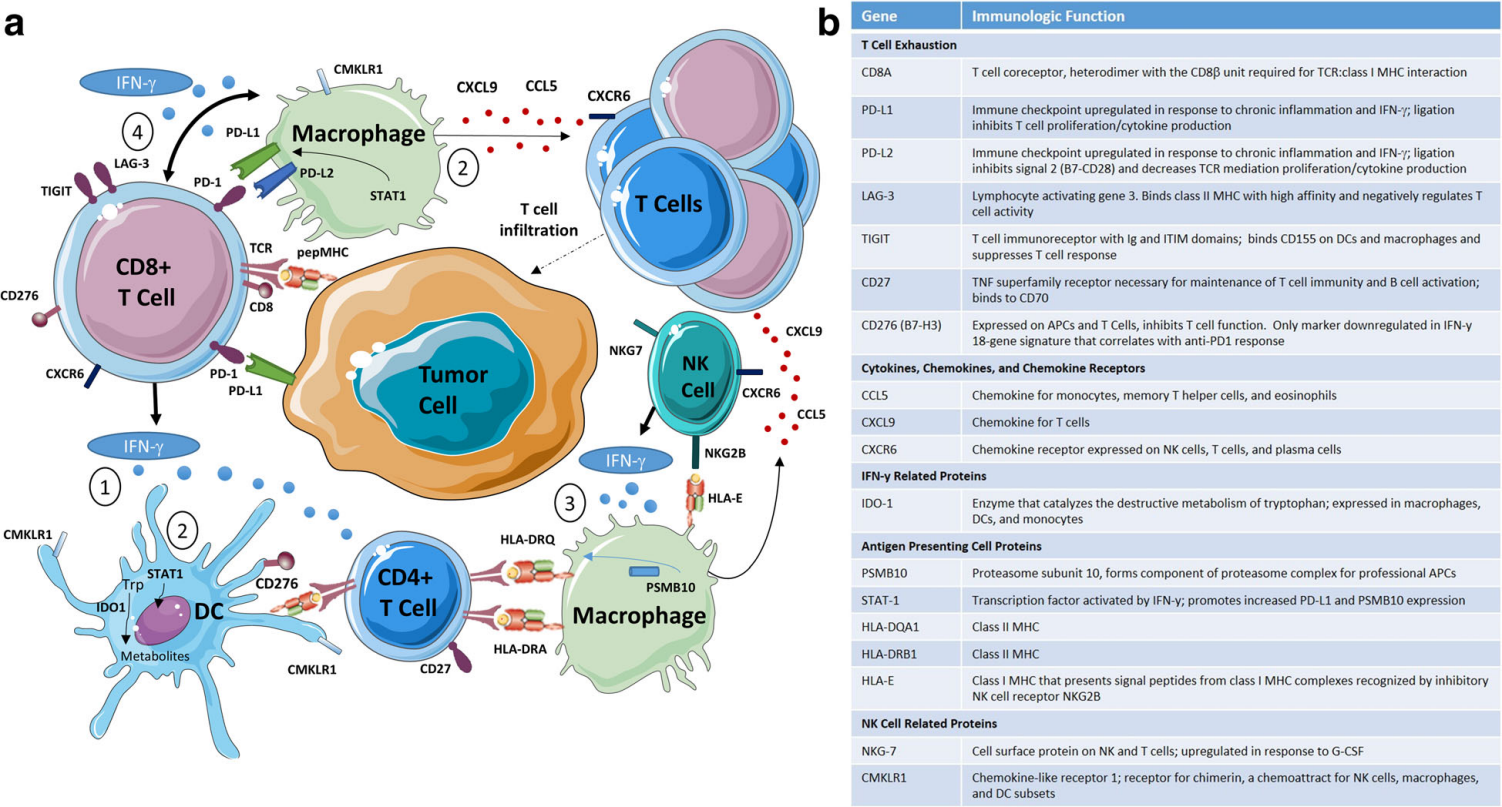
Components of the tumor inflammatory signature as assessed by immune cell gene expression profiling. **a** Complex interplay of chemokines and cytokines classify the inflammatory state of the tumor microenvironment. Interferon-g (IFN-g) released by activated T cells and NK cells activates STAT1, IDO-1 (indolamine oxygenase I) and CMKLR1 in dendritic cells and macrophages (1). STAT-1 mediated signaling and additional pathways produce the chemokines CCL5 and CXCL9 (2). This recruits additional T cells into the tumor microenviroment through CXCR6. IFN-g stimulates the expression of HLA molecules and proteasome components including PSMB10 (3). Finally, IFN-g upregulates a number of immune checkpoint molecules including PD-L1, PD-L2, TIGIT, LAG-3, and B7-H3 on T cells (4). **b** Components of 18-gene immune signature under evaluation in prospective trials with pembrolizumab

An inherent difficulty in using PD-L1 status as a predictive biomarker is that subjective scoring of IHC sections provides information regarding only a single factor in the tumor microenvironment, and doesn’t take into account other features that might more accurately segregate “hot” from “cold” tumors [66, 67]. In that regard, targeted gene expression panels may have the ability to quantify specific RNA expression profiles from formalin-fixed paraffin embedded (FFPE) biopsy and more comprehensively delineate an inflamed tumor microenvironment. An advantage of immune gene expression profiling is that RNA can be quantified from multiple cell types within a specimen which might be more fully representative of the tumor microenvironment (described in Fig. 4a). Immune expression profiling has the potential to accurately determine the inflammatory status of a tumor by quantifying chemokines, cytokines, and cell surface proteins that may better approximate a “hot” tumor than PD-L1 expression alone.

In the Checkmate 275 study with nivolumab in mUC, a 25-gene interferon-gamma (IFN-γ) signature derived from crude extract (EdgeSeq, HTG Molecular Diagnostics Tucson, AZ, USA) was used to assess 177 tumor samples from pretreatment biopsies. Higher values in the IFN-γ gene signature were correlated with response to nivolumab relative to low value IFN-γ expression score (*p* = 0.0003, CR or PR in 20/59 patients with high IFN-γ signature relative to CR or PR in 19/118 patients with medium or low IFN-γ signature). Similar gene expression analysis performed with a chemokine panel showed enrichment in responses from individuals with high expression of CXCL9 and CXCL10 demonstrating the potential to use gene expression profiling as a biomarker. Similar to TMB measurements, the negative predictive value of this gene panel remains problematic as some responses were noted in some patients with a non-inflamed cytokine signature.

Next generation RNA expression technologies allow for immune profiling of greater than 700 genes from isolated RNA. For instance, NanoString nCounter (NanoString Technologies, Seattle, WA) uses 6-color bar codes to identify specific RNA sequencing without gene amplification as is required with traditional RNA sequencing or qPCR technologies. Using a small subset of 19 melanoma patients from Keynote-001, 680 different genes were profiled by Nanostring. A subset of 18 specific genes including interferon-gamma signaling (IDO1 and STAT1), antigen presentation (HLA-DRB1, HLA-DQA1, HLA-E), NK T cell signaling (NKG7, CMKLR1), and additional immunomodulatory proteins (Fig. 4b) were tested in a larger cohort of melanoma patients treated with pembrolizumab in Keynote-001. Validation of the test set in a cohort of patients with head and neck and gastric cancers from the KEYNOTE-028 trial showed a correlation with response to PD-1 therapy, with a deviation of <5% in anti-PD-1 response predictor score [68, 69]. This 18-gene panel has now been validated using tumor specimens from patients across 9 tumor types in 220 patients treated with PD-1 therapy [18] and is currently being evaluated prospectively in 3 ongoing Phase III trials with pembrolizumab (NCT02628067 [70], NCT02559687 [71], and NCT02564263 [72]). The utility of nanostring-based gene expression signatures to predict response to immunotherapy hinges on the results of these pivotal phase III prospective studies – but if successful these data may ultimately guide treatment decisions in mUC and other immunotherapy responsive tumors.

## Conclusions

The FDA approvals of atezolizumab, nivolumab, pembrolizumab, avelumab, and durvalumab represent a major paradigm shift in treating mUC. The recent results of the Phase III IMvigor 211 study, however, suggest the possibility that not all PD-1/PD-L1 reagents will have comparable efficacy. Standardized, reproducible biomarkers (potentially in composite) are needed to accurately guide treatment decisions as no single test has as of yet demonstrated reproducibility to predict responders to immunotherapy. This is of particular importance as there are potentially subgroups of patients with low mutation burden who may respond more favorably to chemotherapy as was noted in Checkmate 026. Although composite biomarkers are of interest, the next generation of predictive biomarkers for immunotherapy might involve either an assessment of tumor mutational burden (TMB) or a targeted gene expression profile with particular attention to T cell gene signatures; these ongoing studies are of critical importance in optimizing precision immunotherapy for patients with metastatic urothelial cancer.

## Abbreviations

AE: Adverse event
CI: Confidence interval
FDA: Food and Drug Administration
IC: Immune cell
IHC: Immunohistochemistry
MSI: Microsatellite instability
mUC: Metastatic urothelial cancer
NSM: Non synonomous mutation
ORR: Objective response rate
OS: Overall survival
PD-1: Programmed cell death protein 1
PD-L1: Programmed death ligand 1
PFS: Progression-free survival
TC: Tumor cell
TCGA: The Cancer Genome Atlas
TMB: Tumor mutation burden
